# Dialysis facility staff perceptions of racial, gender, and age disparities in access to renal transplantation

**DOI:** 10.1186/s12882-017-0800-6

**Published:** 2018-01-10

**Authors:** Kristie J. Lipford, Laura McPherson, Reem Hamoda, Teri Browne, Jennifer C. Gander, Stephen O. Pastan, Rachel E. Patzer

**Affiliations:** 1 Department of Surgery, Division of Transplantation, Emory University School of Medicine, 1639 Pierce Dr. NE, Atlanta, GA 30322 USA; 2 College of Social Work, University of South Carolina, Columbia, SC USA

**Keywords:** Dialysis, Kidney transplant, End stage renal diseases, Barriers, South

## Abstract

**Background:**

Racial/ethnic, gender, and age disparities in access to renal transplantation among end-stage renal disease (ESRD) patients have been well documented, but few studies have explored health care staff attitudes towards these inequalities. Staff perceptions can influence patient care and outcomes, and identifying staff perceptions on disparities could aid in the development of potential interventions to address these health inequities. The objective of this study was to investigate dialysis staff (*n* = 509), primarily social workers and nurse managers, perceptions of renal transplant disparities in the Southeastern United States.

**Methods:**

This is a mixed methods study that uses both deductive and inductive qualitative analysis of a dialysis staff survey conducted in 2012 using three open-ended questions that asked staff to discuss their perceptions of factors that may contribute to transplant disparities among African American, female, and elderly patients.

**Results:**

Study results suggested that the majority of staff (*n* = 255, 28%) perceived patients’ low socioeconomic status as the primary theme related to why renal transplant disparities exist between African Americans and non-Hispanic whites. Staff cited patient perception of old age as a primary contributor (*n* = 188, 23%) to the disparity between young and elderly patients. The dialysis staff responses on gender transplant disparities suggested that staff were unaware of differences due to limited experience and observation (*n =* 76, 14.7%) of gender disparities.

**Conclusions:**

These findings suggest that dialysis facilities should educate staff on existing renal transplantation disparities, particularly gender disparities, and collaboratively work with transplant facilities to develop strategies to actively address modifiable patient barriers for transplant.

## Background

Kidney transplantation (KT) can improve the quality of life for end-stage renal disease (ESRD) patients; [1] however, not all ESRD patients have equal access to transplant [2]. Past studies have indicated gaps in all stages of the transplant process, where racial/ethnic minorities (vs. whites) [3] and females (vs. males) are less likely to access several of the required steps necessary to receive a kidney transplant [4–7]. Evidence also indicates lower likelihood of waitlisting and receiving a deceased or living donor kidney transplant for elderly patients [4, 5, 7, 8]. The new kidney allocation system (KAS), effective as of December 2014, was developed in part to address racial transplant disparities [9, 10]. One of the primary aims of the KAS was to create a more equitable donor system in order to increase deceased donor kidney transplantation and expand access for candidates who have been historically disadvantaged, such as racial minorities [7, 11, 12]. Early results indicate that the KAS is meeting its intended goals of improving access for disadvantaged groups, specifically racial minorities [9]. Nevertheless, there is still room for improvement, especially in increasing access to the steps leading up to kidney transplantation including a) referral from a dialysis facility to the kidney transplant center for medical evaluation, b) starting and completing the required medical and psychosocial evaluation at a transplant center, and c) placement on the national transplant waiting list. [2, 3, 12–14]

Studies have suggested that provider biases (often implicit), limited health disparity knowledge, poor communication, incongruent and culturally incompetent care, and the inability to provide culturally relevant care, are associated with provider contributors to disparities [15–19]. However, few studies have examined the role of healthcare staff on transplantation disparities. Prior research has shown that dialysis staff have the ability to influence patient’s decision making and health behaviors such as completion of advanced directives and adhering to fluid and glycemic control recommendations [20–24]. Moreover, their close and frequent proximity to dialysis patients makes them privy to patients’ needs, allows occasions to advocate on patients’ behalf, and leaves opportunities to shape patients’ medical decision making [22, 23]. Thus, it is likely that the dialysis staff’s knowledge and beliefs regarding transplant access can influence dialysis patients’ decisions about moving ahead with the transplant process. The purpose of this study was to examine dialysis staff perceptions of disparities in renal transplantation. Insights garnered from this study could inform the development of dialysis facility-based efforts to identify and address dialysis staff misperceptions regarding kidney transplant disparities.

## Methods

### Study design

We conducted a cross-sectional survey of all dialysis facilities in ESRD Network 6 (North Carolina, South Carolina, and Georgia) as part of the Reducing Disparities in Access to kidNey Transplantation (RaDIANT) Community Study (R24MD008077; www.clinicaltrials.gov: NCT02092727) [25]. Survey and RaDIANT Community Study methodology have been described elsewhere [26–28], but in brief, medical directors of all dialysis facilities in ESRD Network 6 (*n* = 586) were contacted in 2012 by email and asked to distribute a 25-item electronic survey to the staff member most likely to educate patients about the transplantation process. The inclusion criteria for the study included English fluency, active employment at a dialysis facility, and consent to participate. The survey assessed staff members’ philosophy, attitudes, perceptions, and care behaviors related to transplant access. Ethical approval for this study was obtained by the Institutional Review Board of Emory University and the University of South Carolina.

The secondary, qualitative data analysis reported in this paper focuses on three open ended questions included in the original 25-item survey. Dialysis facility staff were asked three questions about transplant disparities: “Research shows that (1) African Americans, (2) older people, and (3) females with ESRD are less likely to access multiple steps in the kidney transplant process, including referral for transplant evaluation, completion of the kidney transplant evaluation, placement on the national waiting list, and receipt of a living donor (LD) or deceased donor (DD) kidney transplant. In your experience, what factors do you believe contribute to this disparity?”

### Data coding

Respondent answers were analyzed using both a deductive and inductive coding approach. The research team created a short list of a priori codes (poverty, social support, transportation, low education, provider practices) based on a literature review of past studies reporting barriers to kidney transplantation [13, 29–38]. To ensure interrater reliability, two trained research assistants and the investigator (K. Lipford) independently classified all of the providers’ written comments by initially searching for key phrases and statements that could be categorized with the a priori codes. While examining all responses, the research team also inductively coded emergent themes that could not be captured by the deductive coding. We established the criterion for classification if there was agreement between two of the three raters on the classification of each response. Each coder independently examined the comments written by the providers and created relevant themes. When comments were not direct or straightforward, an interpretative approach was utilized. Providers could report more than one factor influencing the specified disparity; we focused our analyses on the frequency of listed factors versus the frequency of responding providers. Thus, each factor listed counted as one unit of analysis. After individually coding, the three coders convened, compared, deliberated, and synthesized the results. Coders met multiple times to discuss the patterns and themes until each response could be appropriately grouped in a category and the team achieved code saturation. Provider non-responses (blanks) were not included in the analyses. Final themes were generated through group consensus.

### Statistical analyses

Raw data from the dialysis facility survey were used for both quantitative and qualitative analyses. Descriptive statistics were used to describe staff and facility characteristics. Staff respondent survey data were linked to dialysis facility demographics in the 2009–2012 Dialysis Facility Report data through facility provider number. Analyses were performed using SPSS 22.0 and SAS 9.4 [39, 40]. Qualitative responses were coded and tallied to calculate frequency count, calculate percentages of total frequencies in each category, and to generate a descriptive statistic, specifically the mode, for each group (African American, elderly, and female).

## Results

Among the 586 dialysis facilities that were contacted and asked to participate, 545 staff members in 509 facilities responded to the survey, yielding an 86.9% facility-level response rate. Most respondents were nurse managers (51%) and social workers (21.8%). The remaining respondents primarily held administrative roles such as facility directors (12.6%) and clinical managers (5.1%). The majority of responding dialysis facilities served a primarily African American (AA) patient population (60.6%), with 43 being the median number of patients that each facility treated [15, 41, 42]. These statistics along with other selected characteristics are described in Table 1.

**Table 1:** Selected Characteristics of Network 6 Dialysis Facilities and Staff Survey, 2012

Facility and Patient Characteristics	Study Population	Georgia	North Carolina	South Carolina	Significance Test^a^
	*n* = 545 Median (IQR)	*n* = 259 Median (IQR)	*n* = 174 Median (IQR)	*n* = 112 Median (IQR)	
*Facility characteristics*					*Test Statistic* ^*b*^
Number of patients per facility, median	43 (30.0, 67.0)	40 (28.0, 63.0)	50 (30.0, 79.0)	45 (33.0, 60.0)	9.65 (<0.001)
Number of staff, median	11 (7.0, 15.0)	9 (7.0, 14.0)	12 (8.0, 17.0)	12 (7.0, 17.0)	11.91 (0.00)
For profit facilities, (%)	87.1	88.4	84.6	87.7	1.36 (0.00)
*Patient characteristics*					
Patient age, median	61 (58.4, 65.0)	61 (58.0, 65.2)	62 (59.2, 65.0)	61 (58.6, 64.0)	1.62 (0.49)
Female, (%) (Mean, SD)	47 (7.7)	47 (8.2)	45 (6.8)	47 (7.5)	2.53 (0.08)
African American, (%)	60 (38.4, 75.0)	61 (35.7, 76.9)	57 (36.3, 74.4)	62 (42.8, 78.9)	5.28 (0.24)
Non-Hispanic white, %	37 (21.0, 57.2)	36 (20.0, 59.2)	40 (22.7, 57.1)	37 (21.0, 55.5)	2.88 (0.50)
Uninsured, (%)	9 (0.0, 16.6)	8 (0.0, 17.6)	8 (0.0, 13.7)	10 (0.0, 18.8)	4.05 (0.28)
Unemployed, (%)	71 (5.8, 100.0)	66 (50.0, 91.6)	71 (57.1, 91.6)	75 (60.0, 100.0)	10.45 (0.05)
Years on dialysis, median	5 (4.2, 5.4)	5 (4.19, 5.5)	5 (4.1, 5.1)	5 (4.4, 5.6)	5.84 (0.05)

^a^The Kruskal-Wallis chi-square test is used for compare GA, NC, and SC

^b^The *p* value is included in parenthesis next to the test statistic

There were 905 total comments made regarding contributors for AA disparities, 842 total comments provided for elderly disparities, and 516 total comments made for female disparities. The gaps in these numbers indicate that staff listed more unique contributing factors for AA disparities and elderly patients than they did for female patients.

### Perceptions of contributors to kidney transplantation disparities for African American ESRD patients

#### Low socioeconomic status

The primary theme related to why renal transplant disparities exist between African American and non-Hispanic whites, reported by dialysis providers, was low socioeconomic status (28.1%). Lack of financial support for post-transplant medications was one of the most prevalent sub-themes that staff members perceived as an issue (*n* = 20, 8.0%). Others stated that many AA patients are unable to draft an adequate financial plan to handle pre- and post-transplant costs that may not be covered by insurance (*n* = 9, 3.9%). Other dialysis providers (*n* = 8, 3.1%) commented that many of their patients do not want to lose their disability income. This fear of ineligibility results in the transplant process becoming a low priority for these patients. Some staff (*n* = 6, 2.3%) believed low socioeconomic status was simply the result of generational poverty among AAs. Other staff (n = 8, 3.0%) expressed that many of their patients fear they will be unable to return to the workforce.

#### Transportation and travel challenges

The second theme related to racial disparities between AA and white ESRD patients was transportation difficulties (16.2%) and the challenge of getting to and from appointments. The cost of travel (*n* = 10, 8.5%), specifically, was a consistent theme and is related to financial challenges. The following comments are representative of the staff comments:

- *In my experience, African Americans are often from low income families that are unable to provide transportation to necessary appointments to complete the evaluation process.*
- *Financial cost of transportation to and from medical appointments.*

Another travel theme was related to healthcare access and the actual distance to transplant centers (*n* = 11, 9.4%), particularly for those patients in rural and micropolitan areas. The following three statements were representative of this theme:

- *Lack of out-of-town transportation for multiple appointments to transplant centers.*
- *Not being able to afford to travel that distance.*
- *Transportation to appointments and evaluations that may be a distance away from their home.*

#### Limited health literacy and lack of education

The third theme representing staff perception of AA disparities was low health literacy and education (8.8%). Some staff even mentioned the need for a liaison or patient navigator that could assist patients and provide more detail about the surgery, post-surgery, medications, and adverse occurrences that medical providers may have trouble relaying to the patient.

- *I think education and awareness are the barriers to transplantation in (the) African American community. The patients need a liaison…all things that the nephrologist, social worker, or dialysis staff are not going to know.*

Some staff even criticized the educational process at facilities. For example, one staff member commented,

- *Individualized transplant education is not provided. Not enough information is given to families. Dialysis staff do not have the time to provide enough transplant education.*

#### Staff disagreement with transplantation disparities

The data also suggests that some staff seemed to disbelieve there were existing racial disparities (4.8%) or were either unsure or unaware of existing disparities (6.6%). Staff who appeared to challenge documented disparities in KT access often did so by referring to lack of evidence in their facility. These comments included,

- *This does not seem to be the case at our clinic...we have an equal number of so called white and so called black patients on transplant list.*
- *We just recently had an African American patient receive a transplant.*
- *We feel that in our facility it isn’t true.*
- *I don’t see this discrimination.*
- *I do not feel there is a disparity of African Americans with ESRD in this facility.*
- *Really not sure that it has been different for African Americans as opposed to White.*

#### Other contributors to AA disparities

Staff also identified several other contributors to racial disparities such as limited social support (7.1%) and low patient adherence (6.6%). Other responses commented on the themes that may discourage patients from progressing with a transplant, such as fear of undergoing surgery and transplant failure of peers (6.4%). One staff member responded,

- *In my own patient population, a lot of patients are afraid of any modality besides HD (hemodialysis) because of ‘bad stories’ from loved ones or friends with kidney failure.*

Cultural beliefs also seemed to play a part in contributing factors (3.7%). Some of the staff responses included ideas about patients’ embrace of providentialism concerning their health and patients’ belief that their faith would provide healing. These ideas are representative of the statements below.

- *Many individuals have very stoic ideas about procedures such as transplant; sometimes there are religious overtones guiding their thinking.*
- *Wishful thinking that kidney will function on its own.*
- *…religious beliefs regarding God going to heal them from the disease*

There also were perceptions of other moral and cultural values (0.38%) that possibly contribute to disparities. For example, one staff member commented that he/she often hears AA patients say, “I can’t stand the thought of somebody else’s body part in mine” which deters patients from moving forward with a transplant. Table 2 presents 20 themes related to racial KT disparities and includes a selected quotation for each theme.

**Table 2:** Identified themes in Dialysis Staff Responses Regarding Racial Disparities (Total Responses = 905)

Themes and Contributing Factors	N (%)	Selected Quotes
*Financial Factors*		
Low Socioeconomic Status	255 (28.1)	*Transplant centers want them to stock pile money for meds post-transplant. How? Donations? Working?? Not enough incentive*
Transportation Challenges	117 (16.2)	*Inability to complete tests due to lack of transportation*
*Clinical Factors*		
Comorbidities	31 (3.4)	*Depression due to disease*
*Patient Factors*		
Lack of Patient Education	80 (8.8)	*Inadequate education about transplant, evaluation, waiting list process*
Lack of Social Support	65 (7.1)	*No suitable partner to help with after care*
Lack of Patient Adherence/Follow Up	60 (6.6)	*Most patients do not follow through with all that is required to complete transplant process*
Patient Fears	58 (6.4)	*Possibility of transplant failure and returning to facility discourages patients from considering the option*
Complexity of Transplant Process	24 (2.6)	*Patients state ‘there is just too much to do, I didn’t know I was gonna have to do all that’*
Lack of Motivation/Interest	19 (2.1)	*Loss of hope appears to turn into loss of interest…*
Lack of Living Donors	7 (0.77)	*Living donors being uneducated*
Content with Existing Care	7 (0.77)	*Outpatient center is a form of socialization*
Refusal to Accept Illness	3 (0.33)	*Denial*
*Cultural Factors*		
Patient Beliefs	34 (3.7)	*Beliefs regarding transplantation - mainly donation of organs – both living and cadaver*
High Medical Mistrust	3 (0.33)	*African Americans have an historical reticence to seeking out medical health or advice*
*Staff and Provider Factors*		
Reasons for Disparity Unknown	60 (6.6)	*I do not have experience in this area; None; Unsure*
Staff Disagreed with Disparities	44 (4.8)	*There is no disparity in this clinic. Race is not a factor when considering transplant for a patient*
Provider Practices	13 (1.4)	*Preconceived stereotyping by staff*
Staff Was Unaware of Disparities	7 (0.77)	*Unaware of disparity, all patients are referred if the patient is interested in transplantation*
*Other Factors*		
Other Reasons	4 (0.44)	*Entitlement attitude???*

### Perceptions of contributors to KT for older ESRD patients

#### Patient age

A major theme related to provider perception of elderly KT disparities was patient awareness of increasing age (22.6%). Within this theme, many staff responded that patients often feel that an available kidney should go to a younger person (*n* = 35, 18.6%), feel as if their advanced age would not be able to tolerate the surgery (*n* = 26, 13.8%), feel they have already lived their life (*n* = 11, 5.8%), do not think they would greatly benefit (*n* = 4, 2.1%), or just simply believe they are “too old” (*n* = 30, 15.9%). Some comments included:

- *I have seen multiple older people who state that they would rather see a younger person with a longer life expectancy get a kidney. We always educate and discourage this thinking. We think that all are deserving of a kidney if the transplant center sees.*
- *Misconception that because of age they would not ever be considered as a viable candidate, that they are wasting their time and energy that the kidney will go to a younger candidate.*
- *Patients feeling as though they have been through enough surgeries and don’t have a much longer life expectancy.*

#### Comorbidities

The second most listed theme was multiple comorbidities (17.0%) which often determines eligibility and placement on the transplant waitlist. Complex comorbidities are associated with graft failure and patient survival post-transplant [43, 44]. Pre-existing conditions can have significant implications before and after surgery. Unfortunately, most staff did not explicitly list the comorbid conditions so we are unsure of what specific diseases they believe may be aiding as contraindications for older ESRD patients in this population. The following two statements are representative of many comments in this category:

- *Older patients have more comorbids and medical conditions holding them back from exploring transplant as an option.*
- *Many of the elder patients have medical problems that will not make them eligible for kidney transplant.*

#### Other contributors to elderly disparities

Similar to the reported contributors of racial disparities, dialysis staff perceived finances to play a major role in the disparity between older and younger ESRD patients. This theme was the third most listed contributing factor (14.3%). Additionally, many staff felt as if patients saw the transplant evaluation process as too complex (4.1%) which leaves many elderly patients uninterested. Some of the complexities cited in this theme include: numerous appointments (38.2%), additional medical testing (29.4), lengthy process (11.7%), complicated follow-up appointments (11.7%), patient difficulty with managing post-transplant medications (8.8%), and lack of comfort navigating the medical system (2.9%). Transportation issues (6.8%) and social support (4.1%) were also cited as reasons contributing to the disparity. As one staff member stated,

- *Difficulty with family members taking off work to transport them since most [elderly patients] no longer drive.*
- *More of older population…have increased barriers to out-of-town transplant centers.*

While transportation issues and social support were reported less often as perceived contributors to disparities in elderly patients, many of the more specific reasons cited by staff, such as lengthy process or difficulty managing post-transplant medications are components contributing to these two all-encompassing themes.

Other perceived barriers for older patients were lack of knowledge concerning transplant center eligibility criteria (0.83%). Staff stated that nephrologists do not refer elderly patients because,

- *They may think they are ineligible.*
- *Lack of updated information on the transplant criteria and who is eligible.*

There were also a few instances where staff explicitly highlighted ageism. For example, the statement below is representative of the age discrimination perceived by dialysis facility staff member,

- *Age is often looked at* vs. *the overall health of the patient [in determining referral].*

Though these types of comments were sparse, they do indicate limited provider knowledge regarding the changes of the KAS. Table 3 describes each coded theme for elderly disparities.

**Table 3:** Identified themes in Dialysis Staff Responses Regarding Age Disparities, (Total Responses = 842)

Themes and Contributing Factors	N (%)	Selected Quotes
*Patient Factors*		
Patient Perceptions of Old Age	188 (22.3)	*Some older people with ESRD feel that they don’t deserve to receive a kidney transplant because of their age and other med*
Lack of Social Support	35 (4.1)	*Not having a stable support system in the home*
Fear	35 (4.1)	*The belief that their heart isn’t strong enough. Fear of “I won’t survive the surgery”*
Lack of Transplant Education	35 (4.1)	*No education on age and how well people do on transplant*
Content with Dialysis	31 (3.6)	*Satisfaction with dialysis as it is easier to incorporate into a retired lifestyle*
No Interest in Transplant	24 (2.8)	*Satisfied with care on a clinical basis*
Time Spent on Waiting List	10 (1.1)	*Patients in this category often feel they will die before they ever get listed*
Burden of/to Family Member	7 (0.83)	*…the desire to not place an additional responsibility on the family would sometimes determine the decision*
Insurance Issues	7 (0.83)	*Insurance constraints*
Misconception about Eligibility	7 (0.83)	*We encourage all patients, but I think older patients are under the impression they are not eligible.*
Lack of Follow-Up	7 (0.83)	*The patient’s primary nephrologist discusses the referral process with the patient and it is the patients’ choice if they want to go through the transplant referral process*
Lack of Living Donors	4 (0.47)	*Lack of living donor options*
*Financial Factors*		
Financial Concerns/Low Socioeconomic Status	121 (14.3)	*Perceived cost of pre-screening appointments*
*Logistical and Transportation Factors*		
Transportation Challenges/Distance to Transplant Center	58 (6.8)	*Difficulty with family members taking off work to transport them since most no longer drive*
*Clinical Factors*		
Comorbidities	144 (17.1)	*By the time they are at end stage their comorbidity list is long*
*Cultural Factors*		
Religious Beliefs	2 (0.23)	*Religious reasons*
Communication Challenges	1 (0.11)	*Communication Issues*
*Staff and Provider Factors*		
Lack of Knowledge about Eligibility	35 (4.1)	*Lack of updated information on the transplant criteria and who is eligible*
Staff Disagreed with Disparities	31 (4.0)	*Again, there is no disparity. We have had 2 patients transplanted with ‘suboptimal’ kidneys due to age*
*Other Factors*		
Complexity of Transplant Process	34 (4.0)	*Lack of comfort when navigating system*
Unknown Reasons	22 (2.6)	*No opinion*
Other Reasons	4 (0.47)	*Comprehension levels*

### Perceptions of contributors to KT for female ESRD patients

#### Limited observations, uncertainty, and lack of knowledge regarding gender disparities

The majority of comments related to female KT disparities describe a lack of observation or experience with gender disparities (14.7%). Many staff members stated that they have *never* had a problem with female patients being less likely to access multiple steps in the kidney transplant process. Some examples of the dialysis staff comments include,

- *There is no disparity in this clinic. Sex is not a factor when considering transplant for a patient.*
- *It’s been my experience my female patients are following through with process.*
- *…female patients are being evaluated at about the same rate as the males in our facility.*

Providers also expressed uncertainty of contributing factors 74 (14.3**%**) which was the second highest category. Most staff simply stated, “don’t know” or “don’t know what causes disparity”. Also, some staff members stated that they were unaware that renal transplant access was an issue for female patients (3.1%). One staff member commented that, “I’m not aware that women are less likely to follow through with referrals”. Others stated,

- *Unaware of the disparity, I didn’t know this was a problem.*
- *I would think that females are more likely to get the process started and complete more so than males.*
- *Unaware of the disparity, I didn’t know this was a problem.*

About 5% of staff comments denied that gender disparities in kidney transplantation exist and roughly 8% clearly stated that there were no known factors that contributed to the disparities. Some of the comments that are representative of the denial of gender gaps include,

- *I have not found this to be true.*
- *Do not agree.*
- *We don’t feel that this is an issue.*

#### Other contributors

Several contributing factors to gender disparities were identified among providers that did recognize a gender disparity in KT. These included a) financial issues (8.7%), b) family obligations (9.3%), c) lack of social support (7.1%), and d) fear of surgery, the surgical process, and/or post-surgery recovery (5.6%). Some comments that were representative of these categories include:

- *Money. The females are often dependent on husbands and/or other family members to meet their financial needs.*
- *Our female patients are more likely to be single/ the caregiver in their support group than our male patients. Male patients typically have a wife or other family member to provide care and support through the process of evaluation and after care.*

Interestingly, 10 (2%) respondents listed body image as a major contributor to the disparity. For example, one staff member responded,

- *In the field of dialysis, I have found that women are most conscious of body image disturbances.*

Weight, presumably being overweight, was also a contributor that was listed by a few staff members. Table 4 details each coded theme along with the frequency and percentage of each.

**Table 4:** Identified themes in Dialysis Staff Responses Regarding Gender Disparities (Total Responses = 512)

Themes and Contributing Factors	N (%)	Selected Quotes
*Patient Factors*		
Family Obligations	48 (9.3)	*…less likely to access steps…due to the demanding nature of dual roles in life (mother, wife, sister, caretaker,* etc.*)*
Lack of Family/Social/Emotional Support	37 (7.1)	*Needing a care partner*
Fear	28 (5.6)	*Fear of who will take care of their families while they are getting the transplant*
Medical Evaluation too Burdensome	22 (4.2)	*The process has too many steps*
Lack of Education/Information	15 (2.9)	*Not understanding the transplant process*
Body Image Concerns	10 (1.9)	*…body image with the impression of heavy scarring after surgery*
Non-Compliance/Lack of Follow-Up	11 (2.1)	*Our main problem is compliance with dialysis and keeping appointments*
Difficulty with Living Donor Inquiries	8 (1.5)	*I think females generally do not want to ‘take’ a kidney from someone else*
Lack of Interest/Motivation	6 (1.1)	*Females seem to be less interested*
Comfortable with Current Treatment	6 (1.1)	*…wanting to stay with what is working for them now (ie. hemodialysis)*
Recovery Time Commitment	4 (0.77)	*Down time for the surgery is a major deterrent*
*Financial Factors*		
Financial Issues	45 (8.7)	*..females are often dependent on husbands and/or other family members to meet their financial needs.*
*Logistical and Transportation Factors*		
Transportation Challenges	17 (3.2)	*Lack of independent transportation*
*Clinical Factors*		
Weight Issues	17 (3.2)	*The challenges of losing weight seem to hinder more women than men in my experience*
*Cultural Factors*		
Religious and Cultural Beliefs	6 (1.1)	*They (females) do not want someone else’s organ in their body*
*Staff and Provider Factors*		
Disparity Not Observed	76 (14.7)	*Haven’t seen this an issue*
Contributing Factors not Known	74 (14.3)	*Not sure about female disparities*
No Contributing Factors	39 (7.5)	*None, have had many female patients receive a transplant*
Staff Disagreed with Disparities	23 (4.4)	*Do not agree*
Unaware of Gender Disparities	16 (3.1)	*Didn’t know this. It’s been my experience my female patients are following through with process*
Discrimination/Gender Bias	4 (0.77)	*Healthcare geared towards men*
Nephrologist Lack of Referral	2 (0.38)	*Lack of referrals*
*Other Factors*		
Other Reasons	2 (0.38)	*Lack of prior experience in acting independently*

## Discussion

Our study explored Southeastern US dialysis facility staff perspectives on the primary contributors to disparities in kidney transplant access. Additionally, the study highlights a few issues that are central to efforts in improving access for vulnerable groups, specifically AA, older, or female ESRD patients. The responses provided by facility staff offer insight into the barriers, complexities, and challenges that both patients and staff face in dealing with ESRD and kidney transplant access.

Two very broad discussions emerge from the themes. The first discussion concerns patient resources. ESRD patient needs often extend beyond traditional medical care such as need for increased social support and transportation. A large proportion of staff highlighted that financial challenges, low social support, lack of education, and limited transportation were major contributors to AA disparities. Furthermore, comorbidities, age, and finances were stressed as the primary contributors for elderly disparities. Financial issues and lack of social support were also cited as main contributors for gender disparities. More work needs to be done in this Network to see if staff members are translating their perceived knowledge regarding transplant disparities into adequate methods to reduce these patient level barriers.

Developing strong community relations is one method to help alleviate health care challenges [45]. Dialysis facilities should consider building relationships with local non-profits, churches, patient advocacy groups, and community clinics due to the tremendous benefit it can have for patients and health delivery. In previous studies, the incorporation of community health workers into the healthcare team led to improvements in diabetes self-management among patients in rural areas [46] and overcoming barriers to medication adherence for hypertension in underserved and diverse patient populations [47]. Moreover, community partners in the non-profit Southeastern Kidney Transplant Coalition organization worked together to develop a multicomponent intervention in Georgia dialysis facilities to reduce racial disparities in referrals for kidney transplant evaluation [48]. A key component in the reduction of KT health disparities are gradual steps toward patient-centered care where the healthcare needs are community-based, personalized, collaborative, and assimilated to the patient [49]. For example, if travel is an issue for patients, do facilities have strategies in place that could help reduce this hurdle for their patients? Also, do dialysis facilities adequately discuss the requirements of KT with their patients and are the providers themselves educated about the KT process? Several of the comments suggested that limited time is devoted to patient education and some respondents felt as if the nephrologists themselves had limited knowledge on the transplant requirements, specifically for older adults. Additionally, recent published literature regarding transplant education has commented on the lack of tailored education [50–54]. If inadequate patient education is a contributor to disparities, one suggestion to remedy this is engaging patients in a peer network support system which has been shown to increase patient education by acting as informational sources [55]. Another effective avenue to improving patient education could be the inclusion of community health workers and/or transplant outreach coordinators whose primary responsibility is to meet patients at dialysis facilities in order to provide answers to transplant related questions. Many of the comments regarding elderly disparities, as well as AA and female disparities, detailed the transplant process being too lengthy, complex, and intimidating for many patients. Community health workers and transplant outreach coordinators can work as adjuncts to health care staff to assist patients with navigating the healthcare system [56]. In summary, if staff perceive and observe patient obstacles in accessing transplantation, more work needs to be devoted to creating partnerships and seeking out community resources that can benefit the patient population they serve and overcoming the reported barriers.

The second discussion that emerges is one of staff bias and limited recognition of disparities. Only a small minority of staff reported on potential biases and discrimination that can work in conjunction with other contributors to disparities. Overall, the majority of staff comments assigned responsibility to the patient (80%) rather than identifying healthcare biases, both individual and institutional, that can exist in the process of accessing a kidney transplant. In fact, a few of the comments mirrored common stereotypes and could be perceived as brash. For example, one staff member stated that “laziness” among AAs was a primary contributor for racial disparities. Undoubtedly, more staff education is needed to discuss ways provider and staff biases can influence patient care and outcomes, and how to overcome these biases [57–60].

Aside from culturally competent and congruent education, more is needed to educate providers on the existence and importance of KT disparities, in general. A primary finding was the lack of knowledge regarding ethnic and female disparities. Clearly, greater awareness and recognition of transplant disparities is required if inequities are to be reduced. A large percentage of staff were not consciously aware, had no suggestions of what contributed to disparities, or completely disagreed that gender transplantation disparities existed. Nearly 5% of staff also denied that racial transplant disparities existed. The denial, low awareness, limited recognition, and lack of suggested contributors, particularly for female and racial disparities, strongly indicates that increased education is necessary to inform staff about gender, racial, and age disparities in kidney transplantation.

### Limitations

Our study is not without limitations. First, only dialysis facilities in ESRD Network 6 were surveyed and our findings may not be generalizable to dialysis staff outside of the Southeastern network. Similar studies are needed to compare and contrast staff in Network 6 to staff perceived barriers to transplantation in other networks, especially since patient characteristics may differ. Second, we did not gather pertinent socio-demographic information on staff such as race and gender which could have helped to further explain some of the nuances with the staff responses. The complex nature of race and gender emphasizes the necessity for researchers to place greater attention on the way the intersection and interconnectedness of patient and provider characteristics influence provider perceptions and actions of care. Third, we did not specify the age characteristics that defined “elderly”, so staff perceptions’ of older patients was likely affected by subjectivity. Despite the limitations, there are noted strengths. Few studies have explored dialysis staff perceptions. This study includes a large sample (*n* = 547) and based on the literature review, it is the largest qualitative data analysis on dialysis staff perspectives. Another strength is the high response rate (93.3%) which makes our results generalizable throughout the Southeast, the region that has the lowest transplant rate and the highest racial transplant disparities in the U.S.

## Conclusion

In summary, our study details dialysis staff perceptions of barriers to kidney transplantation for AA, elderly, and female patients with ESRD. Top staff perceived contributors to disparities in KT access include low socioeconomic status, elderly patients’ beliefs about their age, and lack of experience in dealing with gender transplantation gaps, respectively. Specifically, the majority of staff was unaware and/or failed to acknowledge disparities between male and female ESRD patients. These findings suggest that dialysis facilities should educate staff on existing renal transplantation disparities, particularly gender disparities and collaboratively work with transplant facilities to develop strategies to actively address patient barriers, especially those that are modifiable. Future directions of research should continue to focus on barrier reducing interventions and quality improvement programs in order to reduce these disparities [25, 38, 61–68]. Also, more work is needed to determine how staff perceptions compare with the self-described barriers of patients to transplantation.

## Abbreviations

AA: African American
ESRD: End stage renal disease
KAS: Kidney allocation system
KT: Kidney transplantation
